# Indoor–Outdoor Cats and the “One Health” Perspective: Veterinarian Insight and Influence

**DOI:** 10.3390/vetsci11080330

**Published:** 2024-07-23

**Authors:** Jennifer M. Schoonmaker, Luis Pablo Hervé Claude, Jennifer K. Ketzis

**Affiliations:** 1 One Health Center for Zoonoses and Tropical Veterinary Medicine, Ross University School of Veterinary Medicine, Basseterre KN0101, Saint Kitts and Nevis; schoonmakerjm@gmail.com; 2 Department of Biomedical Sciences, Ross University School of Veterinary Medicine, Basseterre KN0101, Saint Kitts and Nevis; lherveclaude@rossvet.edu.kn

**Keywords:** communication, veterinary resources, veterinary education, rural, human–animal bond

## Abstract

**Simple Summary:**

Allowing cats outdoor access can impact cat health, owner health, and wildlife. However, veterinarian and cat owner discussions about outdoor access can be challenging. This study explored the frequency that veterinarians discussed indoor–outdoor cat topics with clients and the perceived importance of the topics. The most often discussed topics were cat health issues (infectious diseases and trauma) and cat population control. Topics such as zoonoses and impact on wildlife were discussed less often. Cat health issues (diseases and trauma) were also selected by over half of the responding veterinarians as the most import topic when addressing indoor–outdoor cats with clients. The One Health topics “human–animal bond impacts”, “environmental and wildlife impacts”, and “benefits for the cat” were selected as the least important to the veterinarian and client when addressing indoor–outdoor cats. The majority of respondents indicated that they were familiar with One Health. However, only 13% indicated they were extremely comfortable discussing One Health with clients, which might explain the reason for not leveraging One Health topics when addressing indoor–outdoor cats with clients. Not including more One Health topics in discussions is a potentially missed opportunity to educate clients and enable informed decisions.

**Abstract:**

Veterinarian and client discussions about indoor–outdoor cats, although challenging, can impact decisions made regarding cat care and outdoor access. An online survey, exploring topics discussed with clients regarding indoor–outdoor cats and One Health, was made available to veterinarians practicing in the U.S. in 2022. The importance of topics by practice location and to the veterinarian versus what the veterinarian perceived as important to the client were analyzed. Of 280 responding veterinarians, 95% discussed indoor–outdoor cat activities with clients. Cat-associated disease (81%), cat population control (64%), and cat-associated trauma (53%) were discussed most often. With the exception of population control, rural, suburban, and urban veterinarians did not significantly differ in the frequency of topics discussed. Danger to the cat (e.g., trauma and diseases) was the most important topic when addressing indoor–outdoor cats with clients; 57% of veterinarians considered it important to themselves and 61% considered it important to the client. Only 13% of the veterinarians were extremely comfortable discussing One Health with clients and several One Health-related topics (zoonoses and wildlife impacts) were discussed least often. Less focus on One Health topics is potentially a missed opportunity to educate clients, enable more informed client decisions, and improve overall the care of cats.

## 1. Introduction

Keeping cats indoors only versus allowing supervised or unsupervised access to the outdoors is often a controversial topic. While attention to the topic of indoor versus indoor–outdoor cats has increased globally over the last few decades, the risks outdoor cats pose for human and wildlife health were known and published as early as 1916 [1,2]. Indoor–outdoor cats can increase human exposure to zoonotic diseases and infections [3,4,5,6,7,8,9]. They can also affect nature and biodiversity through predation, competition, disease, disturbance, and hybridization [2,10]. In addition to risks to people and wildlife, outdoor cats are more prone to unintentional trauma, disease, and death in comparison to cats kept indoors [11,12]. On the other hand, outdoor cats might contribute to rodent control, have more physical exercise, and fewer behavioral issues [12,13,14]. In New Zealand, 67% of veterinarians thought keeping cats indoor-only would negatively impact their mental health [15]. Also, the impact of outdoor cats on wildlife and biodiversity might be overestimated, given the many other environmental stressors such as climate change, habitat destruction, and pollution [16,17,18].

The indoor versus outdoor cat topic is a significant, complicated One Health issue given the impacts on the cats, public health, the environment and wildlife. There are a wide variety of opinions on cats that spend time outdoors, including variances depending on location (rural, suburban, and urban) and personal cat ownership, with many cat owners allowing outdoor access due to perceived increases in the cat’s well-being and “natural” behaviors [12,13,14,19,20]. Previous studies have assessed some of these differing views, possible factors in decision-making, and current hurdles when addressing indoor–outdoor cats [12,13,18]. In veterinary schools, it is acknowledged that discussing indoor–outdoor cat dilemmas is important and that more veterinarians should discuss this problem; yet, this scenario is not always a reality, and applications to One Health have not been directly studied [20,21].

While there is increasing information on the risks and benefits of indoor–outdoor cats available to cat owners, veterinarians may have an influence on the cat owner’s decision, especially as it pertains to a One Health perspective. Studies have shown that pet owners want their veterinarian’s opinions when discussing their cat’s outdoor access and health and are open to a two-way exchange of information when making decisions about their pet [22,23]. In the study presented herein, veterinarians were surveyed to better understand the subjects discussed in relation to indoor–outdoor cats and their comfort level in discussing One Health when they have conversations about indoor–outdoor cats.

## 2. Materials and Methods

This research was approved by the Ross University School of Veterinary Medicine IRB and used an online questionnaire, developed in Qualtrics (Qualtrics, Provo, UT, USA). After developing the questions and building the survey, seven veterinarians representing different U.S. states and graduation years assessed the questions and tested the survey functionality. Questions were clarified based on feedback. The open survey was advertised via the social media sites of the Colorado Veterinary Medical Association, Association of Avian Veterinarians, and the Ross University School of Veterinary Medicine Alumni Association after obtaining permission from each site. The latter site was selected due to the diversity of states in which members practice as well as the diversity of clinical years and schools they attended. The advertisement included a description of the research, consent information, expected time to complete the questionnaire, investigator contact information, and a link to the survey. The first page of the survey contained consent information and respondents could withdraw consent by exiting the survey at any stage. No personal identifying information was collected. Respondents voluntarily participated with no participation incentives and were a convenience sample. The survey was available from 22 July to 20 November 2022 and standard Qualtrics program measures were used to prevent respondents from taking the survey multiple times.

The survey consisted of 21 questions in three sections: (1) general information and demographics; (2) managing feline cases in practice and indoor–outdoor cat questions regarding appointment conversations; and (3) One Health familiarity and comfort level in discussing One Health (Likert scale) and resources (see Supplemental File S1 for full survey and grouping of questions). Any participants that did not currently practice with cats were directed only to sections (1) and (3). Questions were closed-ended with the ability to fill in “Other” in 7 questions and the option to answer “Prefer not to answer” for 11 questions. Question order was preset, all questions required a response to proceed to the next screen of the survey, and respondents could not go back to previous screens to review responses.

For the purpose of the survey, the definition of “indoor–outdoor cat” was “a domestic cat that is owned and spends some amount of time unsupervised outdoors”. The definition of “One Health” was “an approach that recognized that the health of humans, animals, and the environment are closely connected”.

After the survey was closed, the response data were downloaded into Microsoft^®^ Excel^®^ (version 2311) and responses were reviewed for the inclusion criteria. Those that did not regularly work with cats and those that completed <83% of the survey were excluded from the analysis. Responses were then coded, tabulated, and percentages were calculated as appropriate. Data on the frequency of discussing topics related to indoor–outdoor cats were compared by practice location (rural, suburban, and urban) and data on the importance of topics to the veterinarian and perceived by the veterinarian to be more or less important to the client were compared using a chi-square test (online calculation tools: VassarStats, http://vassarstats.net/ and http://quantpsy.org; last accessed 10 June 2024). None of the calculated percentages or statistical analyses combined response categories. In cases where “prefer not to answer” or “not applicable” were selected, they were omitted from the statistical analysis. To determine if familiarity with the term One Health significantly differed with the year of graduation from veterinary school, a Kruskal–Wallis test was used (Minitab, Minitab^®^, LLC, State College, PA, USA). The significance level utilized for all tests was *p* < 0.05.

## 3. Results

Of the 360 respondents, 63 respondents were excluded due to completing <83% of the survey (21 of which accessed the first page and exited the survey) and 17 respondents did not have any cats as a current part of their caseload. This resulted in 297 respondents with 280 completing most or all questions regarding conversations with clients (Section 2) and 289 completing most or all questions regarding One Health topics (Section 3). While most of the respondents worked in small animal practices (242; 81%), many types of veterinarians were represented including those in mixed practice (19; 6%), academia/research (9; 3%), and government/public health (8; 3%). Most respondents described the location of their practice as suburban (184; 62%), with fewer practicing in urban (64; 22%) and rural areas (44; 15%). Forty-three states, including the District of Columbia, were represented by the respondents. The states with the most responses (>15) were Colorado, Florida, Ohio, Texas, California, and New York (see Supplemental File Table S2 for further demographic data). Most respondents identified themselves as female (251; 85%) and white (264; 89%), with only 3% identifying themselves as members of the Hispanic or Latino community, which aligns with demographics for veterinarians in the U.S.

Of the 280 respondents that see cats as part of their practice, 266 (95%) indicated that they have conversations with clients about indoor–outdoor cats. This conversation was not impacted by the age of the cat, with almost the same number of veterinarians responding “yes” to having had conversations about indoor–outdoor cats with clients when a first visit is completed with a kitten (<6 months old) (228; 83%) or a cat (>6 months old), (227; 83%). The topic most frequently discussed was cat-directed infection or disease (e.g., feline leukemia virus) with 132 (47%) respondents indicating they “always” discussed it with clients and another 94 (34%) respondents indicating they discussed this topic “most of the time” (Table 1). Cat population control, with 125 (45%) of the veterinarians “always” including the topic in client discussions, was the second most common topic. Cat-directed trauma or accidents (e.g., predator attacks, hits by cars, and fights with other cats) were “always” included in the conversation for 63 (23%) of the veterinarians with only 1 veterinarian (<1%) reporting “never” discussing this topic. Of the other topics focused on the cat, outdoor activity was “never” discussed by 49 (18%) respondents and “always” included in the conversation by only 26 (9%) respondents. For health and behavior seen in the home, there was no trend in the frequency that veterinarians discussed the topic; instead, a similar number of responding veterinarians discussed the topic “sometimes”, “about half the time”, “most of the time”, and “always”.

With the exception of cat population control, topics involving One Health issues (possible environmental and wildlife impacts, human–animal bond, and spread and/or possibility of zoonotic disease) were less likely to be included in conversations with clients. Only 34 (12%) respondents “always” discussed possible environmental and wildlife impacts, while 61 (22%) “never” and 108 (39%) only “sometimes” discussed the topic.

Regarding location of the practice (rural, suburban, or urban), there were no statistical differences, based on chi-square tests, between the frequency of discussing a topic and the location with the exception of cat population control (Supplemental File Table S3). The frequency of discussing cat population control differed significantly based on location of the practice (*n* = 278; *p* = 0.006) and was discussed more frequently in rural practices (Table 2).

Most respondents felt that dangers directed towards the cats themselves were the most important topics for the clients (171; 61%) and the veterinarian (160; 57%) (Table 3). The least important topic to the veterinarian and perceived to be for the client was the human–animal bond, followed by the environment and wildlife impact and the benefit for the cat. While there were clear topics that veterinarians considered most important to themselves and the client, there was less consistency in the selection of the other topics. This resulted in statistical differences regarding the topics veterinarians indicated as most important to themselves versus most important to the client (*p* = 0.0002; chi-square). The human–animal bond and the environment and wildlife impact were selected as the least important topics, although the percent for the environment was higher for the client and the percent for the human–animal bond was higher for the veterinarian. This might have led to the statistically significant difference within the least important category (*p* = 0.003; chi-square) (see Supplemental File Table S4).

One Health questions, with 289 respondents, focused on familiarity with the term, comfort level in discussing with clients and colleagues, and sources of information used to learn about One Health. Most respondents (116; 40%) reported that they have an “Average” familiarity with the term “One Health”. Only 34 (12%) rated their familiarity as far above average and 103 (36%) rated their knowledge as somewhat above average. There was a significant difference (*p* = 0.003; Kruskal–Wallis) between graduation year and One Health familiarity with veterinarians who have graduated more recently being more familiar with the term One Health (Table 4).

For comfort level when addressing One Health with clients, 112 (39%) answered “Somewhat comfortable” and 37 (13%) answered “Extremely comfortable”. More respondents reported higher comfort levels when discussing One Health with colleagues, such as other veterinarians and veterinary staff, with 136 (47%) answering “Somewhat comfortable” and 49 (17%) answering “Extremely comfortable” (Table 5).

**Figure 1:**
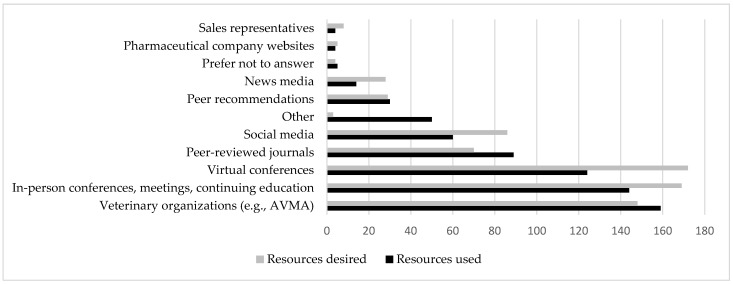
Resources used (*n* = 289) and desired (*n* = 287) by veterinarians for “One Health” information answers in response to the questions “How are you currently learning or have previously learned about ‘One Health?’ (Select up to 3)” and “What resources would be the most helpful for addressing concerns on indoor-outdoor cats and other One Health topics in practice? (Select up to 3)”.

Respondents could choose up to three sources for how they have previously or are currently learning about One Health topics, and most answers included veterinary organizations (159; 23%), in-person conferences and continuing education (144; 21%), virtual conferences (121; 18%), and peer-reviewed journals (89; 13%) (Figure 1). When choosing which resources veterinarians would like to have more of for One Health education, the results were similar in distribution, with most respondents again choosing veterinary organizations (148; 20%), in-person conferences and continuing education (169; 23%), virtual conferences (172; 24%), and peer-reviewed journals (70; 10%). Of the 40 respondents that selected “other” for resources used, 8 respondents indicated no knowledge of One Health, 16 had learned about One Health in veterinary school, 8 had a Master’s in Public Health, and the remainder had used a variety of sources.

## 4. Discussion

This study aimed to explore the conversations veterinarians have with their clients about indoor–outdoor cats, with a focus on One Health topics. While overall, the main topics that the veterinarian considers important versus what the veterinarian perceives the client considers important align, they are not identical and there is a reduced attention on One Health topics compared to those directly impacting the cat’s health.

Based on a study by Foreman-Worsley et al. [13], dangers such as road traffic accidents are a major concern for cat owners. The potential for anthropogenic dangers can be high in rural, urban, and suburban areas, although the specific type of anthropogenic danger can vary by location [9,12,24]. Veterinarians in the survey presented herein ranked this topic the highest for themselves. However, only 53% of respondents “always” or “most of the time” discussed these dangers with clients, and there was no statistically significant difference in the number of veterinarians practicing in rural versus urban or suburban areas regarding this topic of conversation in our study. Despite the anthropogenic dangers, cat owners may still allow outside access due to concerns regarding the cat’s well-being [13]. Nevertheless, 20% of respondents in our study perceived that beneficial activity for the cat was one of the least important topics to discuss with clients, missing an opportunity to address indoor enrichment and other means of ensuring the well-being of indoor-only cats.

Research has demonstrated that the human–animal bond correlates with better pet care and owner satisfaction [25,26]. Also, indoor management for cats has been linked with a greater variety and frequency of care practices such as combing and offering toys, resulting in owners being more prone to having a positive bond with their cats [27]. Despite this fact, most respondents (63%) in our study “never” or only “sometimes” discussed the human–animal bond with clients and selected it as the least important topic for themselves and the cat owner. Possible reasons for veterinarians not directly addressing this topic might include that the term could be ambiguous or that any discussion or care shown for the pets is perceived to already be understood or implied as addressing the human–animal bond. Future studies should include a definition or examples for the human–animal bond to assess if ambiguity or implied inclusion in discussions resulted in its low selection as a topic to address with clients.

Since indoor–outdoor access impacts cats’ health, environmental health, and human health, indicating a One Health concern, veterinarians can use a One Health approach when discussing indoor–outdoor cats with owners [2,6,8,9,12]. There are limited data on veterinarian attitudes towards One Health. However, based on research by Wong and Kogen [28], the vast majority (80%) of veterinary students think that the One Health initiative is very important for public health, such as in food animal medicine, but not as much for equine (23%) or small animal (29%) health. A lack of studies with veterinary practitioners make it unclear if this ranking carries over from school into clinical practice. However, in our study, One Health topics ranked lower in frequency of discussion and importance. Somehow, pet ownership seems to be dissociated from the environmental dimension of One Health, even though the links are evident in the case of a cat’s impact on the environment and wildlife [29]. In our study, the only exception to this was cat population control with rural veterinarians more likely to discuss the topic compared to urban and suburban veterinarians. Concerns about impacts on wildlife or the environment appear removed, however, from population control, since there was no statistically significant difference in the frequency that rural, suburban, and urban veterinarians discussed these topics in our study. It should be noted that other studies have indicated that the level of urbanization is not necessarily associated with people’s connection with nature and wildlife [30,31,32]. Hence, rural veterinarians discussing cat population control might not be driven by One Health issues and the reasons for their focus on this topic require further research.

Work by do Vale et al. [25] has indicated that pet owners depend on veterinarians for public health safety knowledge, with 97% considering the educational role that veterinarians play to be important. Also, a recent survey by Baiyasi et al. [33] indicates that most veterinarians agree that their role is important for zoonotic disease education. However, in our study, zoonotic diseases were an infrequent topic, with 60% of respondents “never” or only “sometimes” discussing these diseases, and therefore contributing to a possible gap between pet owner expectations regarding public health information and the information provided by the veterinarians. The reason for omitting the topic is unclear, although it might be related to the knowledge, skills, and confidence needed for the conversation and fear of negative reception, which are reasons identified as to why difficult conversations are avoided in other healthcare fields [34]. Future research should include evaluating these reasons through more detailed and specific questioning on each individual topic, using surveys and qualitative interviews.

In our study, there was a statistically significant difference in the year of graduation and comfort level in discussing One Health, possibly identifying that veterinary education in One Health has been increasing. This finding matches the attempts to incorporate more One Health education in recent years [35], and One Health knowledge was identified as a core competency for all graduating veterinarians [28,36]. However, our study found it to be a challenge still for some veterinarians to comfortably talk about One Health with their clients. Our study did not assess respondent’s true knowledge of One Health and instead enquired about familiarity with the term to assess if there is an opportunity to provide education regarding indoor–outdoor cat topics within a One Health context. Future studies could explore interactions between One Health knowledge and topics discussed with clients. Understanding the One Health resources veterinarians use and would prefer to use could assist in determining the better contexts in which to provide One Health information to support veterinarians in addressing controversial topics such as indoor–outdoor cats. Specifically, incorporating One Health topics at in-person and virtual conferences and providing One Health resources via professional organizations might help breach the gaps in knowledge and provide veterinarians with the essential skills and confidence needed for these difficult conversations.

A constraint of this study is that the topics addressed were limited and did not explore underlying reasons for the importance placed on the topics and the frequency of the discussions of the topics. In the study presented herein, the most/least important topics to address were assessed over the whole population surveyed. This provided a broad understanding of how these topics were categorized from the veterinarian perspective and the veterinarian’s perceived understanding of the client’s preferences. How frequently an individual veterinarian listed the same topic as important to themself and the client could be included in future studies and to see how this impacts the inclusion or omission of topics. Also, there might have been misunderstandings of the wording by individual respondents, such as for the interpretation of the term “human–animal bond”. There was not a designated question about how the veterinarian personally felt about cats having outdoor access, consultation time constraints, and how they might impact the topics selected for discussion. Further studies should investigate these personal veterinarian opinions and personal practices. Surveys, however, might not be sufficient, in our opinion, to understand some of the nuances in the decisions to include or omit particular indoor–outdoor cat topics, and we suggest that a combination of surveys and qualitative interviews are needed to explore these topics in more depth. Our respondents were practitioners in the U.S. and practitioners elsewhere might prioritize discussion topics differently. However, the topic of indoor–outdoor cats is applicable world-wide with veterinarians facing the same challenging discussions [12,15,20,23].

## 5. Conclusions

In conclusion, based on our findings, there are clear communication gaps and missed opportunities for veterinarians to be educators in discussions with clients about indoor–outdoor cats. Specifically, we found that the responding veterinarians tended to focus most on dangers to the cat and did not leverage One Health topics in their discussions with clients. Future studies could investigate the reasons the veterinarians select certain topics to discuss and how to support veterinarians via continuing education to enable them to better incorporate One Health topics in a clinical setting to address some of the indoor–outdoor cat topics.

## Figures and Tables

**Table 1:** Frequency that veterinarians (*n* = 280) discuss topics with owners of indoor–outdoor cats.

Topic ^1^	Number of Respondents (Percent) ^2^				
	Never	Sometimes	About Half the Time	Most of the Time	Always
Outdoor cat activity	49 (18)	105 (38)	42 (15)	58 (21)	26 (9)
Health and behavior seen in the home	10 (4)	62 (22)	55 (20)	87 (31)	66 (24)
Cat-directed trauma or accidents	1 (0)	95 (34)	37 (13)	84 (30)	63 (23)
Cat-directed infection or disease	2 (1)	32 (11)	20 (7)	94 (34)	132 (47)
Possible environmental and wildlife impacts	61 (22)	108 (39)	40 (14)	37 (13)	34 (12)
Human–animal bond	62 (22)	114 (41)	38 (14)	42 (15)	24 (9)
Spread and/or possibility of zoonotic disease	29 (10)	105 (38)	32 (11)	61 (22)	53 (19)
Cat population control	25 (9)	57 (20)	21 (8)	52 (19)	125 (45)

^1^ The survey question was “How frequently do you address these topics with clients who own indoor-outdoor cats?”. ^2^ Percentages across rows might add to 99 or 101 due to rounding and omitting decimals.

**Table 2:** Frequency that veterinarians (*n* = 280) discussed cat population control by practice location.

Practice Location ^2^	Number of Respondents (Percent) ^1^				
	Never	Sometimes	About Half the Time	Most of the Time	Always
Rural	2 (5)	2 (5)	4 (10)	11 (27)	22 (53)
Suburban	18 (10)	32 (18)	14 (8)	28 (16)	83 (47)
Urban	5 (8)	22 (35)	2 (3)	13 (21)	20 (32)
Other	0 (0)	1 (50)	1 (50)	0 (0)	0 (0)

^1^ Percentages across rows might add to 99 or 101 due to rounding and omitting decimals. ^2^ Frequency of discussing population control significantly differed by location (*p* = 0.006; chi-square test).

**Table 3:** Veterinarians’ (*n* = 280) opinions on the importance of topics regarding indoor–outdoor cats to the veterinarian and the client.

Number of Respondents Selecting Each Category (Percent) ^2^									
Importance ^1^		Benefit for the Cat	Change in Cat Health/Behavior	Danger to the Cat	Environment and Wildlife Impacts	Human–Animal Bond Impacts	Risks of Disease to Owners	Cat Population Control	NA ^3^
Most	Client	26 (9)	30 (11)	171 (61)	6 (2)	2 (1)	25 (9)	18 (6)	2 (1)
	Veterinarian	8 (3)	21 (8)	160 (57)	18 (6)	1 (<1)	47 (17)	24 (9)	1 (<1)
Least	Client	56 (20)	6 (2)	1 (<1)	65 (23)	78 (28)	11 (4)	54 (19)	9 (3)
	Veterinarian	58 (21)	6 (2)	2 (1)	42 (15)	110 (39)	10 (4)	26 (9)	26 (9)

^1^ The survey questions were “Which of these topics do you feel is the most and least important as a veterinarian when addressing indoor-outdoor cats with clients?” and “Which of these topics do you feel is the most and least important to the client when addressing indoor-outdoor cats?”. ^2^ Percentages across rows might add to 99 or 101 due to rounding and omitting decimals. ^3^ Not all veterinarians answered these questions with several selecting “prefer not to answer/not applicable”.

**Table 4:** Veterinarian familiarity with the term One Health (*n* = 289).

How Would You Rate Your Familiarity with the Term “One Health”?				
Far below Average	Somewhat below Average	Average	Somewhat above Average	Far above Average
Number of respondents (percent) ^1^				
19 (7)	17 (6)	116 (40)	103 (36)	34 (12)
Median graduation year (range of graduation years) for selected familiarity ^2^				
2007 (1987–2022)	2008 (1988–2020)	2012 (1985–2022)	2014 (1983–2022)	2015 (1982–2021)

^1^ Percentages across rows might add to 99 or 101 due to rounding and omitting decimals. ^2^ There was a significant difference (*p* = 0.003; Kruskal–Wallis) between graduation year and One Health familiarity.

**Table 5:** Veterinarian comfort level with discussing One Health (*n* = 289).

	Number of Respondents (%)				
How Comfortable Are You When Discussing One Health	Extremely Un-Comfortable	Somewhat Un-Comfortable	Neutral	Somewhat Comfortable	Extremely Comfortable
with clients?	17 (6)	33 (11)	90 (31)	112 (39)	37 (13)
with colleagues?	14 (5)	27 (9)	63 (22)	136 (47)	49 (17)

## Data Availability

Data are available on request from the corresponding author.

## Supplementary Materials

The following supporting information can be downloaded at https://www.mdpi.com/article/10.3390/vetsci11080330/s1, File S1: Questionnaire; File S2: Demographic data; File S3: Responses by location; File S4: Importance by individual respondent.
