# Supplementary material for: Indoor–Outdoor Cats and the “One Health” Perspective: Veterinarian Insight and Influence
Source: Vet Sci. 2024 Jul 23;11(8):330. doi: 10.3390/vetsci11080330 (PMC11359215; doi:10.3390/vetsci11080330)
Supplement: Supplementary file 1 [file vetsci-11-00330-s001.zip › vetsci-3076540-supplementary.pdf]

Supplemental File S1. Questionnaire

Cover letter / Letter posted to websites

Researchers at Ross University School of Veterinary Medicine are inviting veterinarians to take part in a questionnaire about cats and One Health. These data can then be used to assess if there are resources that can benefit veterinarians and/or obstacles being faced that could be remediated.

The questionnaire will take about 15 minutes to complete.

There are no known risks to participating in this study. Your response to the survey is anonymous which means no names will appear or be used on research documents or be used in presentations or publications. The research team will not know that any information you provided came from you, nor even whether you participated in the study.

We hope to receive completed questionnaires from at least 200 participants, so your answers are important to us. Of course, you have a choice about whether or not to complete the survey, and if you do participate, you are free to skip any questions or discontinue at any time.

If you have questions about the study, please feel free to ask; my contact information is given below. If you have complaints, suggestions, or questions about your rights as a research volunteer, contact the IRB Administrator at Ross University School of Veterinary Medicine at RUSVMIRB@rossvet.edu.kn

To continue to the survey web page, follow this link: [link]

Or copy and paste the following URL into your internet browser: [URL]

If you have questions about the study, please feel free to ask; my contact information is given below. If you have complaints, suggestions, or questions about your rights as a research volunteer, contact the IRB Administrator at Ross University School of Veterinary Medicine at RUSVMIRB@rossvet.edu.kn

Thank you in advance for your assistance.

Sincerely,

Dr. Jennifer Schoonmaker
Ross University School of Veterinary Medicine
PHONE: redacted
E-MAIL: jenniferschoonmaker@students.rossu.edu

First page of the questionnaire

Researchers at Ross University School of Veterinary Medicine are inviting veterinarians to take part in a questionnaire about cats and One Health. These data can then be used to assess if there are resources that can benefit veterinarians and/or obstacles being faced that could be remediated.

The questionnaire will take about 15 minutes to complete.

There are no known risks to participating in this study. Your response to the survey is anonymous which means no names will appear or be used on research documents or be used in presentations or publications. The research team will not know that any information you provided came from you, nor even whether you participated in the study.

We hope to receive completed questionnaires from at least 200 participants, so your answers are important to us. Of course, you have a choice about whether or not to complete the survey, and if you do participate, you are free to skip any questions or discontinue at any time.

If you have questions about the study, please feel free to ask; my contact information is given below. If you have complaints, suggestions, or questions about your rights as a research volunteer, contact the IRB Administrator at Ross University School of Veterinary Medicine at RUSVMIRB@rossvet.edu.kn

To continue to the survey, please press the next button.

If you elect not to participate, please exit this page.

Dr. Jennifer Schoonmaker Ross University School of Veterinary Medicine PHONE: redacted E-MAIL: jenniferschoonmaker@students.rossu.edu.

Survey screen 1

1. Please choose the one that BEST describes your current position:

- Small Animal Practice
- Large Animal Practice
- Mixed Animal Practice
- Government/Public Health
- Industry/Corporate
- Academia/Research
- Exotic/Wildlife/Zoological Practice
- Prefer not to answer
- Other ____

2. In what state is your primary location of practice? __________________

3. In which year did you graduate from veterinary school? please use a four number format (e.g., 1999, 2021). ___________________

4. Which best describes your primary location of practice?

- Rural
- Suburban
- Urban
- Prefer not to answer
- Other ____

5. With which gender do you associate yourself?

- Male
- Female
- Non-binary / third gender
- Prefer not to say

6. Please indicate your ethnicity

- African American or Black
- American Indian or Alaska Native
- Asian
- Hispanic or Latino
- Native Hawaiian or Pacific Islander
- White
- Prefer not to say
- Other ____

Survey screen 2

7. Does your caseload currently include domestic cats?
- o Yes, at least one a week
- o Yes, at least one a month but less than one a week
- o Yes, less than one a month
- o No

(Respondent who answered "no" to question 7 automatically skipped to questions 22-25.)

Survey screen 3

8. What is the most commonly used medical resource in your practice to help manage feline cases? Select all that apply
- o Plumbs or other drug formulary
- o Veterinary Information Network (VIN) or other professional website
- o Social Media inclusive of veterinary Facebook groups
- o Textbooks/Journals
- o Webinars/Conferences (continuing education)/Proceedings or Notes
- o Peer recommendations
- o Not applicable, I do not frequently use specific references
- o Other ____

Survey screen 4

For the following questions, indoor-outdoor cat is defined as "A domestic cat that is owned and spends some amount of time unsupervised outdoors."

9. Do you have conversations with clients regarding indoor-outdoor cats?

o Yes   o No

10. When a client brings in a **kitten (<6 months old)** for a first exam with your practice, do you discuss if the cat will be indoor only, indoor-outdoor, or outdoor only?

o Yes   o No   o Sometimes

11. When a client brings in a **cat (6 months old or older)** for a first exam with your practice, do you discuss if the cat will be indoor only, indoor-outdoor, or outdoor only?

o Yes   o No   o Sometimes

Survey screen 5

12. How frequently do you address these topics with clients who own indoor-outdoor cats?

	Never	Sometimes	About half the time	Most of the time	Always
Cat-directed trauma or accidents (predator attacks, hit by-car, fights with other cats, etc.)	◻	◻	◻	◻	◻
Cat-directed infection or disease (feline leukemia virus, fleas, etc.)	◻	◻	◻	◻	◻
Cat population control	◻	◻	◻	◻	◻
Health and behavior seen in the home (inappropriate urination, obesity, etc.)	◻	◻	◻	◻	◻
Human-Animal bond	◻	◻	◻	◻	◻
Outdoor cat activity (physical exercise, mental stimulation, etc.)	◻	◻	◻	◻	◻
Possible environmental and wildlife impacts (bird predation, disease, disturbance, etc.)	◻	◻	◻	◻	◻
Spread and/or possibility of zoonotic disease (Toxoplasma, Giardia, Rabies, etc.)	◻	◻	◻	◻	◻

13. Which of these topics do you feel is the **most important for the client** when addressing indoor-outdoor cats with clients?

- o Beneficial activities for the cat (exercise, enrichment, etc.)
- o Changes in health and behavior (inappropriate urination, obesity, etc.)
- o Dangers directed towards the cats themselves (trauma, disease, etc.)
- o Environmental and wildlife impacts (predation, disease, disturbance, etc.)
- o Human-animal bond impacts
- o Risks of disease to owners (Toxoplasma, Giardia, rabies, etc.)
- o Stray and indoor-outdoor cat population control
- o Prefer not to answer/Not applicable

14. Which of these topics do you feel is the **least important for the client** when addressing indoor-outdoor cats with clients?

- o Beneficial activities for the cat (exercise, enrichment, etc.)
- o Changes in health and behavior (inappropriate urination, obesity, etc.)
- o Dangers directed towards the cats themselves (trauma, disease, etc.)
- o Environmental and wildlife impacts (predation, disease, disturbance, etc.)
- o Human-animal bond impacts
- o Risks of disease to owners (Toxoplasma, Giardia, rabies, etc.)
- o Stray and indoor-outdoor cat population control

- Prefer not to answer/Not applicable

15. Which of these topics do you feel is the **most important as a veterinarian** when addressing indoor-outdoor cats with clients?
   - Beneficial activities for the cat (exercise, enrichment, etc.)
   - Changes in health and behavior (inappropriate urination, obesity, etc.)
   - Dangers directed towards the cats themselves (trauma, disease, etc.)
   - Environmental and wildlife impacts (predation, disease, disturbance, etc.)
   - Human-animal bond impacts
   - Risks of disease to owners (Toxoplasma, Giardia, rabies, etc.)
   - Stray and indoor-outdoor cat population control
   - Prefer not to answer/Not applicable

16. Which of these topics do you feel is the **least important as a veterinarian** when addressing indoor-outdoor cats with clients?
   - Beneficial activities for the cat (exercise, enrichment, etc.)
   - Changes in health and behavior (inappropriate urination, obesity, etc.)
   - Dangers directed towards the cats themselves (trauma, disease, etc.)
   - Environmental and wildlife impacts (predation, disease, disturbance, etc.)
   - Human-animal bond impacts
   - Risks of disease to owners (Toxoplasma, Giardia, rabies, etc.)
   - Stray and indoor-outdoor cat population control
   - Prefer not to answer/Not applicable

Survey screen 6

For the following questions, One Health is defined as "An approach that recognizes that the health of humans, animals, and the environment are closely connected."

17. How would you rate your familiarity with the term "One Health"?
   - Far below average
   - Somewhat below average
   - Average
   - Somewhat above average
   - Far above average

Please select the most appropriate for each statement:

18. How comfortable are you when discussing One Health with **clients**?

Extremely uncomfortable	Somewhat uncomfortable	Neither comfortable nor uncomfortable	Somewhat comfortable	Extremely comfortable
□	□	□	□	□

19. How comfortable are you when discussing One Health with **colleagues**? (Staff, other veterinarians)

Extremely uncomfortable	Somewhat uncomfortable	Neither comfortable nor uncomfortable	Somewhat comfortable	Extremely comfortable
◻	◻	◻	◻	◻

20. How are you **currently learning or have previously learned** about One Health? (Select up to 3)

- Peer-reviewed journals
- Peer recommendations
- Pharmaceutical company websites
- Virtual conferences, meetings and/or continuing education (CE)
- In-person conferences, meetings and/or continuing education (CE)
- News media
- Social media
- Sales representatives
- Veterinary organizations (e.g., AVMA)
- Prefer not to answer
- Other ____

21. What resources **would be the most helpful** for addressing concerns on indoor-outdoor cats and other One Health topics in practice? (Select up to 3)

- Peer-reviewed journals
- Peer recommendations
- Pharmaceutical company websites
- Virtual conferences, meetings and/or continuing education (CE)
- In-person conferences, meetings and/or continuing education (CE)
- News media
- Social media
- Sales representatives
- Veterinary organizations (e.g., AVMA)
- Prefer not to answer
- Other ____

Supplemental File S2. Demographic data, 297 responding veterinarians

Gender	Number of respondents	Percent
Male	41	14
Female	251	85
Non-binary / third gender	3	1
Prefer not to say	2	1

Ethnicity	Number of respondents	Percent
African American or Black	1	0
American Indian or Alaska Native	2	1
Asian	6	2
Hispanic or Latino	10	3
Native Hawaiian or Pacific Islander	0	0
White	264	89
Prefer not to say	11	4
Other	3	1

Practice type	Number of respondents	Percent
Small Animal Practice	242	81
Large Animal Practice	4	1
Mixed Animal Practice	19	6
Government/Public Health	8	3
Industry/Corporate	4	1
Academia/Research	9	3
Exotic/Wildlife/Zoological Practice	5	2
Prefer not to answer	0	0
Other	6	2

Other: feline only, hospice, shelter medicine, government racetrack veterinarian, avians

Practice location	Number of respondents	Percent
Rural	44	15
Suburban	184	62
Urban	64	22
Prefer not to answer	0	0
Other	5	2

Year of graduation	Number of respondents	Percent
1982	1	0.3
1983	1	0.3
1984	1	0.3
1985	1	0.3
1986	2	0.7
1987	3	1.0
1988	1	0.3
1989	3	1.0
1990	3	1.0
1991	7	2.4
1992	2	0.7
1994	4	1.3
1995	2	0.7
1996	2	0.7
1997	4	1.3
1998	5	1.7
1999	1	0.3
2001	5	1.7
2002	6	2.0
2003	6	2.0
2004	6	2.0
2005	9	3.0
2006	9	3.0
2007	10	3.4
2008	9	3.0
2009	8	2.7
2010	11	3.7
2011	12	4.0
2012	12	4.0
2013	16	5.4
2014	16	5.4
2015	15	5.1
2016	30	10.1
2017	22	7.4
2018	13	4.4
2019	16	5.4
2020	9	3.0
2021	9	3.0
2022	5	1.7

State / District of practice	Number of respondents	Percent
Alabama	1	0.3
Alaska	0	0.0
Arizona	4	1.3
Arkansas	1	0.3
California	17	5.7
Colorado	37	12.5
Connecticut	5	1.7
Delaware	2	0.7
District of Columbia (DC)	3	1.0
Florida	26	8.8
Georgia	6	2.0
Hawaii	0	0.0
Idaho	1	0.3
Illinois	11	3.7
Indiana	4	1.3
Iowa	1	0.3
Kansas	4	1.3
Kentucky	2	0.7
Louisiana	3	1.0
Maine	0	0.0
Maryland	11	3.7
Massachusetts	10	3.4
Michigan	7	2.4
Minnesota	3	1.0
Mississippi	0	0.0
Missouri	1	0.3
Montana	1	0.3
Nebraska	0	0.0
Nevada	2	0.7
New Hampshire	4	1.3
New Jersey	12	4.0
New Mexico	2	0.7
New York	18	6.1
North Carolina	8	2.7
North Dakota	2	0.7
Ohio	21	7.1
Oklahoma	0	0.0
Oregon	4	1.3
Pennsylvania	8	2.7
Rhode Island	2	0.7
South Carolina	1	0.3
South Dakota	2	0.7

State / District of practice	Number of respondents	Percent
Tennessee	6	2.0
Texas	20	6.7
Utah	0	0.0
Vermont	2	0.7
Virginia	9	3.0
Washington	3	1.0
West Virginia	1	0.3
Wisconsin	1	0.3
Wyoming	0	0.0
Other	1	0.3
Prefer not to answer	7	2.4

Supplemental File S3. Responses by location

Response to the Question: "How frequently do you address these topics with clients who own indoor-outdoor cats?" based on Primary location of Practice (n = 280)

Topic	Never	Sometimes	About half the time	Most of the time	Always
	Number of respondents (percent)				
Outdoor cat activity					
Rural (N=41)	4 (10)	17 (41)	4 (10)	14 (34)	2 (5)
Suburban (N=175)	30 (17)	63 (36)	28 (16)	35 (20)	19 (11)
Urban (N=62)	15 (24)	23 (37)	10 (16)	9 (15)	5 (8)
Other (N=2)	0 (0)	2 (100)	0 (0)	0 (0)	0 (0)
Health and behavior seen in the home					
Rural	0 (0)	12 (29)	10 (24)	12 (29)	7 (17)
Suburban	9 (5)	34 (19)	28 (16)	59 (34)	45 (26)
Urban	1 (2)	16 (26)	16 (26)	15 (24)	14 (22)
Other	0 (0)	0 (0)	1 (50)	1 (50)	0 (0)
Cat-directed trauma or accidents					
Rural	0 (0)	15 (36)	9 (22)	11 (27)	6 (15)
Suburban	0 (0)	58 (33)	20 (11)	53 (30)	44 (25)
Urban	1 (2)	22 (35)	8 (13)	18 (29)	13 (21)
Other	0 (0)	0 (0)	0 (0)	2 (100)	0 (0)
Cat-directed infection or disease					
Rural	0 (0)	6 (15)	2 (5)	16 (39)	17 (41)
Suburban	1 (<1)	20 (11)	13 (7)	54 (31)	87 (50)
Urban	1 (2)	6 (10)	5 (8)	22 (35)	28 (45)
Other	0 (0)	0 (0)	0 (0)	2 (100)	0 (0)
Environmental and wildlife impacts					
Rural	8 (20)	17 (41)	4 (10)	9 (22)	3 (7)
Suburban	40 (23)	64 (37)	26 (15)	20 (11)	25 (14)
Urban	13 (21)	26 (42)	10 (16)	7 (11)	6 (10)
Other	0 (0)	1 (50)	0 (0)	1 (50)	0 (0)
Human-Animal bond					
Rural	6 (15)	19 (46)	8 (20)	5 (12)	3 (7)
Suburban	39 (22)	69 (39)	22 (13)	28 (16)	17 (10)
Urban	17 (27)	25 (40)	7 (11)	9 (15)	4 (6)
Other	0 (0)	1 (50)	1 (50)	0 (0)	0 (0)

Topic	Never	Sometimes	About half the time	Most of the time	Always
	Number of respondents (percent)				
Spread/possibility of zoonotic disease					
Rural	5 (12)	14 (34)	6 (15)	8 (20)	8 (20)
Suburban	17 (10)	63 (36)	19 (11)	40 (23)	36 (20)
Urban	7 (11)	27 (43)	6 (10)	13 (21)	9 (15)
Other	0 (0)	1 (50)	1 (50)	0 (0)	0 (0)
Cat population control					
Rural	2 (5)	2 (5)	4 (10)	11 (27)	22 (53)
Suburban	18 (10)	32 (18)	14 (8)	28 (16)	83 (47)
Urban	5 (8)	22 (35)	2 (3)	13 (21)	20 (32)
Other	0 (0)	1 (50)	1 (50)	0 (0)	0 (0)

Location versus frequency of discussing cat population control was statistically significant based on a chi-square test: p = 0.006. For all other topics, based on chi0square tests, the frequency of discussing did not differ by practice location: cat-directed trauma or accidents p = 0.333; cat-directed infection or disease p = 0.960; health and behavior seen in the home p = 0.269; the human-animal bond p = 0.638; outdoor cat activity p = 0.427; possible environmental and wildlife impacts p = 0.811); and spread and/or possibility of zoonotic disease p = 0.708).

Supplemental File S4. Importance by individual respondent

In the study presented, topic of most/least importance was compiled over all the respondents. Agreement between the topic an individual veterinarian selected for themself versus selected for the client was not assessed. A stand-alone analysis of this should also consider reasons for the selection (knowledge, complexity, controversy, etc.). These data are, therefore, presented as supplementary to the manuscript but should be interpreted with caution.

Topic	Most important		Least important	
	Number of veterinarians that selected the topic for themselves	Number of veterinarians that selected the topic for themselves and clients (percent)	Number of veterinarians that selected the topic for themselves	Number of veterinarians that selected the topic for themselves and clients (percent)
Dangers directed towards the cats themselves (trauma, disease, etc.)	154	110 (71)	2	1 (50)
Risks of disease to owners (Toxoplasma, Giardia, rabies, etc.)	45	12 (27)	10	1 (10)
Stray and indoor-outdoor cat population control	23	9 (39)	24	13 (54)
Changes in health and behavior (inappropriate urination, obesity, etc.	21	9 (43)	5	2 (40)
Environmental and wildlife impacts (predation, disease, disturbance, etc.)	18	3 (17)	42	23 (55)
Beneficial activities for the cat (exercise, enrichment, etc.)	7	2 (29)	53	28 (53)
Human-animal bond impacts	1	0	109	56 (51)
Total agreement	269	145 (54)	245	124 (51)

Answers to the questions "Which of these topics do you feel is the most and least important as a veterinarian when addressing indoor-outdoor cats with clients?" and "Which of these topics do you feel is the most and least important to the client when addressing indoor-outdoor cats?"
Not all respondents answered all four questions.
